# Supplementary material for: Whey Cheeses Containing Probiotic and Bioprotective Cultures Produced with Ultrafiltrated Cow’s Whey
Source: Foods. 2024 Apr 16;13(8):1214. doi: 10.3390/foods13081214 (PMC11048827; doi:10.3390/foods13081214)
Supplement: Supplementary file 1 [file foods-13-01214-s001.zip › foods-2935354-supplementary.pdf]

Table S1. Physichochemical parameters of whey cheese samples. Average values and standard deviation (±).

	DRY MATTER		
Storage time (days)	C	LA5	LA5FQ4
1	16.83 ± 0.36 a,A	19.50 ±0.77 a,B	18.82 ± 0.28 a,b,B
7	18.66 ± 0.59 b,A	19.90 ± 0.25 a,B	20.61 ± 0.34 b,B
14	19.23 ± 0.33 b,A	21.46 ± 3.10 a,A	20.04 ± 1.82 a,b,A
21	18.11 ± 0.36 b,A	19.44 ± 0.43 a,B	19.25 ± 0.52 a,b,B
28	16.07 ± 0.51 a,A	19.04 ± 1.42 a,B	18.10 ± 0.71 a,A,B
	aw		
Storage time (days)	C	LA5	LA5FQ4
1	0.934 ± 0.00 a,A	0.936± 0.00 a,b,A	0.943 ± 0.00 b,c,B
7	0.937 ± 0.00 c,A	0.944 ± 0.00 c,A,B	0.946 ± 0.00 c,B
14	0.938 ± 0.00 c,A	0.939 ± 0.00 b,c,A	0.940 ± 0.00 b,A
21	0.937 ± 0.00 b,c,A	0.938 ± 0.00 b,c,A	0.939 ± 0.00 b,A
28	0.935 ± 0.00 a,b,B	0.932 ± 0.00 a,A	0.933 ± 0.00 a,A
	pH		
Storage time (days)	C	LA5	LA5FQ4
1	6.67 ± 0.06 b,C	5.27 ± 0.06 a,b,A	5.80 ± 0.10 d,B
7	6.63 ± 0.06 b,B	5.37 ± 0.06 b,c,A	5.43 ± 0.06 a,b,A
14	6.93 ± 0.06 c,C	5.47 ± 0.06 c,A	5.67 ± 0.06 c,d,B
21	6.53 ± 0.15 b,B	5.27 ± 0.06 b,A	5.23 ± 0.06 a,b,A
28	6.17 ± 0.12 a,C	5.07 ± 0.06 a,A	5.47 ± 0.12 b,c,B
	TITRATABLE ACIDITY		
Storage time (days)	C	LA5	LA5FQ4
1	0.035 ± 0.01 a,A	0.208 ± 0.01 a,C	0.164 ± 0.01 a,B
7	0.089 ± 0.00 b,A	0.431 ± 0.01 c,C	0.399 ± 0.01 d,B
14	0.100 ± 0.01 b,A	0.570 ± 0.02 d,C	0.246 ± 0.02 b,B
21	0.101 ± 0.00 b,A	0.378 ± 0.00 b,C	0.261 ± 0.01 b,B
28	0.176 ± 0.01 c,A	0.451 ± 0.01 c,C	0.332 ± 0.02 c,B

Different superscript letters (a,b,c,d) indicate differences for the same product in different ripening days. Superscript capital letters (A,B,C) indicate differences between products at the same ripening day.

Table S2. CIEL*a*b* parameters of whey cheese samples. Average values and standard deviation (±).

	L*		
Storage time (days)	C	LA5	LA5FQ4
1	86.90 ± 1.49 a,b,B	87.23 ± 1.79 a,B	82.00 ± 0.35 a,A
7	82.17 ± 2.07 a,A	87.10 ± 3.20 a,A,B	91.17 ± 3.20 b,B
14	83.90 ± 3.56 a,b,A	87.00 ± 2.76 a,A	87.13 ± 2.37 a,b,A
21	88.00 ± 2.82 a,b,A	86.70 ± 0.61 a,A	87.90 ± 2.51 a,b,A
28	89.83 ± 0.51 b,B	90.43 ± 1.85 a,B	84.23 ± 2.29 a,A
	a*		
Storage time (days)	C	LA5	LA5FQ4
1	-3.43 ± 0.21 a,B	-2.57 ± 0.23 a,A	-2.8 ± 0.26 a,A

7	-3.07 ± 0.32 ^{a,A}	-3.50 ± 0.40 ^{b,c,A}	-2.93 ± 0.06 ^{a,A}
14	-3.20 ± 0.62 ^{a,A}	-3.77 ± 0.15 ^{c,A}	-3.13 ± 0.31 ^{a,A}
21	-2.43 ± 0.06 ^{a,A}	-2.57 ± 0.45 ^{a,A}	-2.70 ± 0.17 ^{a,A}
28	-2.70 ± 0.40 ^{a,A}	-2.63 ± 0.32 ^{a,b,A}	-3.10 ± 0.17 ^{a,A}
	b*		
Storage time (days)	C	LA5	LA5FQ4
1	6.27 ± 0.55 ^{a,A}	5.80 ± 0.20 ^{a,A}	6.53 ± 1.17 ^{a,A}
7	7.83 ± 0.06 ^{a,A,B}	8.87 ± 0.80 ^{c,B}	7.50 ± 0.40 ^{a,A}
14	8.13 ± 1.95 ^{a,A}	8.80 ± 0.30 ^{c,A}	6.87 ± 0.45 ^{a,A}
21	6.77 ± 0.29 ^{a,A}	6.97 ± 0.51 ^{a,b,A}	6.77 ± 0.64 ^{a,A}
28	6.17 ± 0.15 ^{a,A}	7.30 ± 0.26 ^{b,B}	7.40 ± 0.52 ^{a,B}

Different superscript letters (a,b,c) indicate differences for the same product in different ripening days. Superscript capital letters (A,B) indicate differences between products at the same ripening day.

Table S3. Microbiological counts of whey cheese samples. Average values and standard deviation (±).

	LACTOCOCCUS		
Storage time (days)	C	LA5	LA5FQ4
1	6.46 ± 0.14 ^{a,A}	8.18 ± 0.10 ^{a,B}	8.81 ± 0.03 ^{b,C}
7	7.90 ± 0.03 ^{b,A}	8.53 ± 0.21 ^{a,B}	8.08 ± 0.11 ^{a,A}
14	8.51 ± 0.34 ^{c,A}	9.47 ± 0.12 ^{b,B}	9.33 ± 0.18 ^{c,B}
21	8.71 ± 0.16 ^{c,A}	8.80 ± 0.03 ^{a,A}	9.21 ± 0.01 ^{c,B}
28	8.32 ± 0.31$^{b, c,A}$	8.21 ± 0.39 ^{a,A}	9.06 ± 0.06 ^{b,c,B}
	LACTOBACILLUS		
Storage time (days)	C	LA5	LA5FQ4
1	5.49 ± 0.02 ^{a,A}	8.27 ± 0.08 ^{a,B}	8.89 ± 0.01 ^{b,C}
7	5.96 ± 0.20 ^{a,A}	8.33 ± 0.21 ^{a,B}	8.21 ± 0.26 ^{a,B}
14	7.03 ± 0.38 ^{b,A}	9.36 ± 0.20 ^{b,B}	9.19 ± 0.04 ^{b,B}
21	7.23 ± 0.53 ^{b,A}	8.44 ± 0.33 ^{a,B}	9.19 ± 0.09 ^{b,C}
28	8.43 ± 0.62 ^{d,A}	8.48 ± 0.25 ^{a,A}	9.18 ± 0.07 ^{b,B}
	YEASTS AND MOULDS		
Storage time (days)	C	LA5	LA5FQ4
1	4.75 ± 0.35 ^{a,A}	5.74 ± 0.25 ^{a,B}	5.27 ± 0.24 ^{a,b,A,B}
7	4.10 ± 0.20 a,A	5.93 ± 0.40 ^{a,B}	5.73 ± 0.51 ^{a,b,B}
14	6.61 ± 0.44 ^{b,c,A}	6.24 ± 0.95 ^{a,A}	6.27 ± 0.25 ^{b,A}
21	7.46 ± 0.14 ^{c,B}	5.65 ± 0.27 ^{a,A}	5.02 ± 1.04 ^{a,A}
28	6.48 ± 0.37 ^{b,B}	6.72 ± 0.34 ^{a,B}	5.74 ± 0.30 ^{a,b,A}

Different superscript letters (a,b,c,d) indicate differences for the same product in different ripening days. Superscript capital letters (A,B,C) indicate differences between products at the same ripening day.
